# Micronutrient Sprinkles to Control Childhood Anaemia

**DOI:** 10.1371/journal.pmed.0020001

**Published:** 2005-01-25

**Authors:** Stanley H Zlotkin, Claudia Schauer, Anna Christofides, Waseem Sharieff, Mélody C Tondeur, S. M. Ziauddin Hyder

## Abstract

Over 750 million children have iron-deficiency anemia. A simple powdered sachet may be the key to addressing this global problem

## Teaser

A simple powdered sachet may be the key to addressing a global problem

Recent World Health Organization (WHO)/United Nations Children's Fund estimates suggest that the number of children with iron-deficiency anaemia (IDA) is greater than 750 million [1]. Iron deficiency is the most common preventable nutritional problem despite continued global goals for its control. Historically, the problem of IDA in children largely disappeared in North America when foods fortified with iron and other micronutrients became available for children. In this group, the prevalence of IDA has fallen from 21% in 1974 to 13% in 1994 [2]. Although pockets of infants and children remain at risk, generally, the eradication of iron deficiency in developed countries is recognized as a successful public health accomplishment. This solution has not worked in developing countries where commercially purchased fortified foods are not available or are not used.

In the developing world, there are three major approaches available to address iron deficiency: dietary diversification so as to include foods rich in absorbable iron, fortification of staple food items (such as wheat flour), and the provision of iron supplements. When dietary or fortification strategies are not logistically or economically feasible, supplementation of individuals and groups at risk is an alternative strategy. For the past 150 years or more, oral ferrous sulphate syrups have been the primary strategy to control IDA in infants and young children [3]. However, adherence to the syrups is often limited owing to a combination of their unpleasant metallic aftertaste, the dark stain they leave on the child's teeth, and abdominal discomfort [4]. Thus, despite the ongoing work of the United Nations Standing SubCommittee on Nutrition and others to solve the problem of poor adherence in infants and young children, all interventions to date have been universally unsuccessful [1,5]. In this article, we describe our efforts, stage by stage, towards achieving the goal of controlling IDA.

## The Strategy

Our research group at the Hospital for Sick Children in Toronto conceived the strategy of “home fortification” with “Sprinkles”—single-dose sachets containing micronutrients in a powdered form, which are easily sprinkled onto any foods prepared in the household. We hypothesized that this would be a successful method to deliver iron and other micronutrients to children at risk [6]. The idea of Sprinkles was formulated in 1996, when a group of consultants determined that the prevention of childhood IDA was a United Nations Children's Fund priority, yet available interventions (syrups and drops) were not effective [7].

In Sprinkles, the iron (ferrous fumarate) is encapsulated within a thin lipid layer to prevent the iron from interacting with food. This means that there are minimal changes to the taste, color, or texture of the food upon adding Sprinkles. Other micronutrients including zinc, iodine, vitamins C, D, and A, and folic acid may be added to Sprinkles sachets. Any homemade food can be fortified with the single-dose sachets, hence the term “home fortification”. Two formulations have been developed, a nutritional anaemia formulation (Table 1) and a complete micronutrient formulation (Table 2). 

**Table 1:**
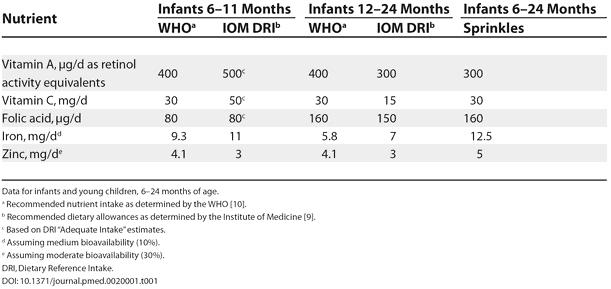
Daily Dose and Derivation of Sprinkles Nutritional Anaemia Formulation for Home Fortification of Complementary Foods

Data for infants and young children, 6–24 months of age

^a^ Recommended nutrient intake as determined by the WHO [10]

^b^ Recommended dietary allowances as determined by the Institute of Medicine [9]

^c^ Based on DRI “Adequate Intake” estimates

^d^ Assuming medium bioavailability (10%)

^e^ Assuming moderate bioavailability (30%)

DRI, Dietary Reference Intake

**Table 2:**
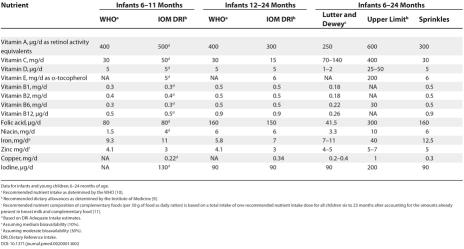
Daily Dose and Derivation of Sprinkles Complete Micronutrient Formulation for Home Fortification of Complementary Foods

Data for infants and young children, 6–24 months of age

^a^ Recommended nutrient intake as determined by the WHO [10]

^b^ Recommended dietary allowances as determined by the Institute of Medicine [9]

^c^ Recommended nutrient composition of complementary foods (per 50 g of food as daily ration) is based on a total intake of one recommended nutrient intake dose for all children six to 23 months after accounting for the amounts already present in breast milk and complementary food [11]

^d^ Based on DRI Adequate Intake estimates

^e^ Assuming medium bioavailability (10%)

^f^ Assuming moderate bioavailability (30%)

DRI, Dietary Reference Intake

## Clinical Trials

### Efficacy

To investigate the bioavailability of the iron in Sprinkles, we used a dual stable isotope method and showed that anaemic infants absorbed iron from Sprinkles about twice as efficiently as nonanaemic infants when delivered in a maize-based diet in West Africa. The study was conducted in collaboration with the Kintampo Health Research Centre of the Ministry of Health in Accra, Ghana. The geometric mean iron absorption from two doses of iron (30 mg and 45 mg of elemental iron per sachet) was 8.3% (range, 2.9%–17.8%) in infants with anaemia and 4.5% (range, 1.1%–10.6%) in infants without anaemia [8]. Comparing these absorption values to the new American/Canadian Dietary Reference Intake standards for infants, we concluded that during infancy (i) iron absorption of Sprinkles from a maize-based porridge met and surpassed needs for absorbed iron, and (ii) iron absorption is up-regulated in infants with IDA [9,10,11]. Based on these results, we estimated through computer simulations that a 12.5-mg iron dose (as recommended by the WHO) from Sprinkles should be adequate for use in large-scale distribution programs for the prevention and treatment of mild to moderate anaemia.

It has been suggested that zinc may compete with iron for the same receptor sites on intestinal mucosal cells in the proximal duodenum, thereby compromising the absorption of both minerals [12]. To address this important issue, we recently conducted a bioavailability study in rural Ghana using the same dual stable isotope method as previously used [8]. In order to determine the effect of two doses of zinc on the absorption of iron from Sprinkles (with 30 mg of elemental iron), 63 young children, 12–24 months of age with varying haemoglobin levels were studied. We found that 10 mg of zinc (in the form of zinc gluconate) added to Sprinkles significantly reduced the absorption of iron, whereas a 5-mg dose had no effect. Thus, we concluded that adding 5 mg of zinc to the formulation of Sprinkles was appropriate (unpublished data).

Over the past five years, we have completed seven community-based trials in four different countries [13,14,15,16,17,18,19]. The goal of these studies was to test the efficacy of Sprinkles in diverse settings. When we pooled data from two of our studies that compared Sprinkles to the reference standard, ferrous sulphate drops, we had a total of 518 anaemic infants (haemoglobin < 100 g/l) who were given one of two ferrous sulphate doses (15 mg or 40 mg of elemental iron as ferrous sulphate) and 318 similar infants who received one of four doses of iron from Sprinkles (12.5 mg, 20 mg, 30 mg, or 80 mg of elemental iron as microencapsulated ferrous fumarate) [13,18]. This gave us greater than 97% power (α = 0.05) to detect whether the mean difference in the end-of-study haemoglobin concentrations between ferrous sulphate and Sprinkles regimens was within ± 5 g/l (a range of equivalence). Using a random effects model (for study and dose) that adjusted for baseline haemoglobin, we found no significant difference between Sprinkles and drops.

**Figure 1:**
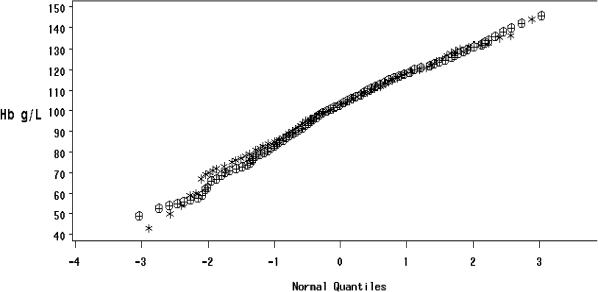
Overlaid Quantile-Quantile Plots of Haemoglobin Concentrations at the End of Studies for Sprinkles and Ferrous Sulphate Drops The graph shows that the two distributions overlap at all quantiles, thus proving that there is an equivalent response to the two treatments for haemoglobin concentrations. Circles represent individuals who received iron drops; crosses represent individuals who received Sprinkles.

We further examined this through quantile-quantile plots of haemoglobin concentrations at the end of the studies for Sprinkles and ferrous sulphate drops (Figure 1). The overlaid plots of haemoglobin concentrations of the Sprinkles and drops groups show that these two distributions overlap at all quantiles. These plots clearly indicate that the haemoglobin response to the two different forms of iron was equivalent. Thus, we have concluded that Sprinkles are as efficacious as the current reference standard for the treatment of anaemia. Overall, 55%–90% of the anaemic infants who were provided with Sprinkles were cured.

### Acceptability

During our studies we also asked about the caregivers' perception of their infants' responses to Sprinkles as compared to drops, the Sprinkles' impact on the food to which they were added (change in taste, color, or consistency), the use of sachets as a delivery vehicle, and the perceived side effects of Sprinkles [13,18,19]. Invariably, the response to Sprinkles has been positive. No appreciable change in the food with Sprinkles has been reported, no one reported stains on the infants' teeth, and Sprinkles were reported to be easy to use. The only consistently reported side effect was a darkening of the infant's stool, which is expected since most of the iron is excreted in stool. In a recently conducted study in Bangladesh, using a four-point measurement scale, 60% of the mothers “extremely liked”, 30% “liked”, and the remaining 10% “somewhat liked” the Sprinkles intervention; no one disliked Sprinkles. Major reasons cited for liking Sprinkles included ease in mixing Sprinkles with complementary (i.e., weaning) foods and that their use promoted the appropriate introduction of complementary foods, since Sprinkles could be used only if complementary foods were used [19].

## Ensuring a Sustainable Supply

As the results of the first studies showing the efficacy of Sprinkles became available, the need for a reliable high-quality supply became apparent. In 2000, the H. J. Heinz Company of Pittsburgh, Pennsylvania, United States, expressed an interest in the Sprinkles program as a component of their corporate social responsibility program. Since 2001, the H. J. Heinz Company has provided support and expertise in the evaluation of consumer needs and a supply of Sprinkles for research, while the H. J. Heinz Company Foundation has provided financial support for research activities. Through a formal process of technology transfer, local overseas Sprinkles production has been encouraged. Currently, an independently licensed copacker is supporting local production for a national program in Guyana, and plans are in place for technology transfer to Bangladesh and Pakistan.

## Scaling Up for Countrywide Distribution

The final stage, the scale-up process, is by far the most challenging. First, this process involves dialogue with the Ministries of Health, scientific community, civil society, and other private partners. Second, it is important to identify sustainable methods of distribution that are able to reach and provide Sprinkles to the most vulnerable populations in the developing world. From our experience in Mongolia, we have determined that it is feasible to distribute Sprinkles in partnership with a non-governmental organization called World Vision. Sprinkles sachets distributed in Mongolia over a two-year period included both iron and vitamin D. Sprinkles have been successfully distributed by World Vision field staff to over 15,000 children in seven districts. Coverage has been over 80%, at a cost of about US$0.03 per sachet. In the project area, the prevalence of anaemia (haemoglobin < 115 g/l) and rickets decreased from 42% to 24% and 48% to 33%, respectively [20].

Notwithstanding these positive results on anaemia control, without committed, long-term financial input from national governments, international agencies, or nongovernmental organizations, sustainability is not guaranteed. Clearly, sustainability over the long term can most likely be achieved if a program becomes self-financing. This may be achieved through public- and private-sector partnerships that use effective social marketing models or possibly through programs which include microcredit in order to reach poorer population groups.

When strategizing how to scale up Sprinkles from small-scale research projects to large-scale programs, we quickly realized that our research group did not have the necessary funding, experience, or personnel needed to influence health policy, develop a social marketing strategy, or maintain a distribution network at a countrywide level. We have thus partnered with organizations that specialize in each of these areas to help achieve our goal of sustainable distribution.

For example, the government of Pakistan is planning to distribute Sprinkles through their ongoing Lady Health Worker Program, which is the largest public-sector primary health-care program implemented by the Federal Ministry of Health. In Bangladesh, BRAC (formerly known as Bangladesh Rural Advancement Committee), the largest national non-governmental organization in the country, is planning to distribute Sprinkles through their ongoing Female Community Health Worker program (popularly known as Shastha Shebika). In both of these countries, Sprinkles would be produced locally through public–private partnerships via a technology transfer agreement. The cost per sachet of locally produced Sprinkles should range from US$0.010 to US$0.015, depending on the volume of production, as compared to US$0.020 to US$0.025 if imported.

## Conclusion

Each stage in the evolution of the Sprinkles intervention has been evaluated in a controlled manner. We determined that the use of encapsulated iron did not appreciably change the taste or color of the food to which it was added, we showed that the haemoglobin response in anaemic infants was equivalent to the current standard of practice, and we documented the acceptability of Sprinkles among caregivers who used Sprinkles in their homes. Finally, through various partnerships, we have developed a successful model to scale up the intervention for countrywide use. Our challenge for the future is to demonstrate the cost-effectiveness of this new intervention and to advocate for the adoption of Sprinkles in the nutrition policy of developing countries.

## Abbreviations

IDA: iron-deficiency anaemia
WHO: World Health Organization
